# Cold‐inducible RNA‐binding protein contributes to intracerebral hemorrhage‐induced brain injury via TLR4 signaling

**DOI:** 10.1002/brb3.1618

**Published:** 2020-04-13

**Authors:** Kai Zhou, Shengtao Cui, Wei Duan, Jianrong Zhang, Jiacheng Huang, Li Wang, Zili Gong, Yu Zhou

**Affiliations:** ^1^ Department of Neurology Xinqiao Hospital & The Second Affiliated Hospital The Army (Third Military) Medical University Chongqing China; ^2^ Graduate School The Army (Third Military) Medical University Chongqing China

**Keywords:** cold‐induced RNA‐binding protein, inflammation, intracerebral hemorrhage, Toll‐like receptor 4

## Abstract

**Introduction:**

Excessive neuroinflammation aggravates the brain injury caused by intracerebral hemorrhage (ICH), while the upstream mechanisms that initiate neuroinflammation remain unclear. Toll‐like receptor 4 (TLR4) signaling is important to trigger inflammatory responses in ICH, and cold‐inducible RNA‐binding protein (CIRP) has been shown as a novel ligand of TLR4 by recent studies. However, whether the CIRP could trigger the neuroinflammation via activating TLR4 signaling in ICH still needs to be investigated.

**Methods:**

Human serum CIRP levels were measured using the ELISA kits. Western blot, FJB staining, brain water content, and neurological deficit scores were used to investigate the roles of CIRP in brain injury caused by ICH.

**Result:**

First, we found increased CIRP levels in the blood of patients with ICH when compared to the control individuals, and the ICH patients with mRS > 2 have higher serum CIRP levels in contrast to those with mRS ≤ 2. In the ICH mice, we also found that brain CIRP protein and mRNA levels were also increased after ICH. Furthermore, using the CIRP^−/−^ mice, we found that CIRP^−/−^ mice had less brain damages showing in less FJB^+^ cells, reduced brain water content (BWC) and lower neurological deficit scores (NDS) compared to that in WT mice after ICH. Cytokines including IL‐6, TNF‐α, and IL‐1β from CIRP^−/−^ mice were attenuated after ICH. CIRP^−/−^ mice also exhibited reduced TLR4 expression which was accompanied by the decreased activity of NF‐κB. This suggests that TLR4 signaling might be involved in CIRP‐mediated inflammatory injury possibly via NF‐κB activation after ICH.

**Conclusion:**

Our findings suggest that CIRP may activate TLR4 signaling, and further inducing NF‐κB activation to increase the expression levels of cytokines and aggravate inflammatory injury in ICH. Targeting CIRP may be a promising strategy for ICH treatment.

## Graphical abstract

Understanding the upstream mechanisms that initiate neuroinflammation has the potential to identify targets for intracerebral hemorrhage (ICH) treatment. Our data indicate that CIRP may activate TLR4 signaling to increase the expression levels of cytokines and aggravate inflammatory injury in ICH. This study provides a novel insight into the initial events that triggering neuroinflammation after ICH and a new therapeutic target for ICH treatment.

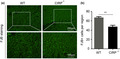

## SIGNIFICANCE STATEMENT

Understanding the upstream mechanisms that initiate neuroinflammation has the potential to identify targets for ICH treatment. Our data first found increased serum CIRP levels were negatively correlated to the outcomes of patients with ICH. Then, we used the mouse ICH model to investigated the roles of CIRP in triggering neuroinflammation, and the results indicate that CIRP may activate TLR4 signaling to increase the expression levels of cytokines and aggravate inflammatory injury in ICH. This study provides a novel insight into the initial events that triggering neuroinflammation after ICH and a new therapeutic target for ICH treatment.

## 1 INTRODUCTION

Intracerebral hemorrhage (ICH) could result in high mortality and morbidity, while still lacking of effective medical therapies to improve outcomes in patients with ICH (Xiong, Liu, & Yang, 2016; Zhou, Wang, Wang, Anne Stetler, & Yang, 2014). Increasing evidence has shown that excessive neuroinflammatory responses contribute to the ICH‐induced brain injury (Zhou, Wang, et al., 2014). The damage‐associated molecular patterns (DAMPs), such as heme (Lin et al., 2012), hemoglobin (Hb; Wang et al., 2014), and HMGB1 (Ohnishi et al., 2011), released by the damaged neural cells, and have been shown to trigger the neuroinflammation mainly to aggravate brain injury after stroke (Zhou, Wang, et al., 2014). Although interfering these DAMPs could significantly reduce the inflammatory injury after ICH, there are still higher levels of inflammation compared to the control group, which further suggesting that there may be existed some other DAMPs released by damaged neural cells led to the activation of neuroinflammation after ICH.

Cold‐inducible RNA‐binding protein (CIRP) is a 172‐amino acid nuclear cold shock protein. The intracellular CIRP has been shown to be involved in regulating cellular physiological processes, such as cell growth, senescence, and apoptosis (Zhu, Buhrer, & Wellmann, 2016), which may be related to the modulation of the post‐transcriptional level of genes (Morf et al., 2012). Besides, the extracellular CIRP has recently been considered as a DAMP to trigger inflammatory responses during hemorrhagic shock and sepsis (Qiang et al., 2013). Although CIRP has been shown to constitutively but weakly expressed in some tissues, like brain, it would markedly be upregulated in ischemia stroke (Zhou, Yang, Ji, Qiang, & Wang, 2014). These results indicated that CIRP released by damaged neural cells stimulates inflammation to cause neuronal damage.

Furthermore, DAMPs have been shown to trigger neuroinflammation via Toll‐like receptors (TLRs), like TLR4. CIRP, one of the DAMPs, also activates inflammatory cells to induces a cytotoxic profile in a TLR4‐dependent manner to promote the inflammatory response in sepsis (Bolognese et al., 2018; Qiang et al., 2013). These research data strongly suggested that CIRP may also act as a DAMP to involve in the regulation of neuroinflammation and aggravate the inflammatory injury after ICH.

Therefore, according to the abovementioned information, we speculated that CIRP would contribute to neuroinflammation via triggering TLR4 signaling and lead to brain damage after ICH. In this study, we first explored the expression profile of CIRP and its role in brain injury caused by ICH. Then, we further explored the underlying mechanisms of CIRP on regulating inflammation in mediating neuronal injury after ICH.

## 2 MATERIALS AND METHODS

### 2.1 Patients data

To evaluate the relationship between CIRP and outcomes of patients with ICH, 34 ICH patients and 20 healthy controls (age and sex matched) were enrolled in our study. The inclusion criteria of ICH patients were as follows: (a) ages from 18 to 80 years; (b) the diagnoses were made following the criteria of the European Stroke Initiative and the 4th National Conference on Cerebrovascular Diseases (1996); (c) primary ICH within 24 hr from onset; (d) basal ganglia hemorrhage confirmed by brain CT scan; and (e) in agreement with the study protocol. The exclusion criteria were as follows: (a) sepsis within the last 1 year; (b) in a coma or death within 48 hr after being admitted by hospital; (c) hemorrhage caused by coagulation abnormalities, brain cancer, trauma, vascular malformations, anticoagulation treatment, or drug abuse; (d) evident inflammatory or autoimmune diseases, like rheumatoid arthritis, systemic lupus erythematosus (SLE), or infectious disease; (e) the presence of nosocomial infection; (f) hepatopathy; and (g) diabetes mellitus. After admitting to the hospital, the patients were asked to draw 5 ml cubital vein blood, and serum was obtained after 3,000 *g* centrifugation for 20 min. Adopted the modified Rankin Scale (mRS) scores to assess ICH patients' neurologic deficits at 3 months after onset. The blood samples of all the enrolled participates were collected with their informed consents, and the research procedures were approved by the Ethics Committee of Xinqiao Hospital of Army (Third Military) Medical University (Chongqing, China) and implemented in accordance with the Helsinki Declaration and its amendments.

### 2.2 Generation of *CIRP* knockout mice and experimental animals

The *CIRP* knockout mice were generated according to the previous reported methods (Yang et al., 2013). Briefly, two sgRNAs were designed to target a region upstream of exon 1 and downstream of the exon 7, respectively. Different concentrations of Cas9 mRNA and sgRNAs were blended and co‐injected into the cytoplasm of one‐cell stage fertilized eggs to produce chimeras. PCR genotyping and sequencing indicated that some pups carried deletions of about 10 kb spanning two sgRNA target sites removing the CIRP exon 1‐7. The generation of *CIRP* knockout mice was conducted by Beijing Biocytogen Co. Ltd.

The CIRP^−/−^ mice had C57BL/6 background. Male C57BL/6 mice (20–25 g) were purchased from Daping Hosptial, Army (Third Military) Medical University. All mice were housed in a clean and temperature‐controlled environment on a 12 hr light‐to‐dark cycle and free access to water and food. All animal experiments were performed followed by the Guide for the Care and Use of Laboratory Animals, 8th edition (2011). All the animal experiments were approved by the Animal Ethics Committee of Xinqiao Hospital, Army (Third Military) Medical University.

### 2.3 Enzyme‐linked immunosorbent assay (ELISA) measurement

The human plasma CIRP levels were measured following the manufacturer's instructions of CIRP (CUSABIO). For the measurement of the IL‐1β, IL‐6, and tumor necrosis factor‐α (TNF)‐α levels of perihematomal brain tissue of ICH mice, the perihematomal brain tissues should first be homogenized into supernatants. Briefly, clearing the blood of perihematomal brain tissues in precooled PBS (0.02 mol/L, pH 7.0–7.2). Then, after weighing, the tissue and precooled PBS (mass/volume ratio: 1:5) were mixed and homogenized using the Homogenizer. The obtained homogenate was further treated by ultrasonic crushing, then centrifuged at 5,000 *g* for 5 min to obtain the supernatant for detection. Then, following the manufacturer's instructions, the inflammatory factors IL‐1β and IL‐6 and tumor necrosis factor‐α (TNF)‐α levels of these supernatants were further detected by using IL‐1β, IL‐6, and TNF‐α (Dakewe Biotech Company) ELISA reagent kits, respectively.

### 2.4 ICH models

Autologous blood ICH model was used in this study (Xiong, Liu, Wang, et al., 2016). According to the previous reported methods, we constructed the ICH models. Briefly, the mice were fixed on a mouse stereotaxic frame (RWD Life Science Co) after anesthetizing by 80 mg/kg phenobarbital injection via intraperitoneal (i.p.). Then, 20 μl of autologous blood collected from the tail vein was directly injected into the striatum (at 0.8 mm anterior, 2 mm right lateral, and 3.5 mm deep from the bregma) using a syringe pump (KD Scientific).

### 2.5 Quantitative real‐time PCR

The total RNA was extracted from the perihematomal brain tissue at 12 hr, and 1, 3, 5, and 7 days after ICH using TRIzol reagent (Invitrogen). RT‐PCR was operated according to the manufacturer's instructions (Takara Biotechnology), and β‐actin was adopted for internal control. The sequence of primers used in PCR is as follows: CIRP forward: 5′‐AGCTCGGGAGGGTCCTACAG‐3′ and reverse: 5′‐GAGGGCTTTTACTCGTTGTGTGT‐3′; β‐actin: forward: 5′‐ CGTGAAAAGATGACCCAGATCA‐3′ and reverse: 5′‐ TGGTACGACCAGAGGCATACAG‐3′. The 2^−△△^CT method was used to calculate the relative mRNA expression levels.

### 2.6 Western blot

According to previous reported methods (Yang et al., 2017), the SDS‐PAGE was used to analysis the proteins from perihematomal brain tissues at 12 hr, and 1, 3, 5, and 7 days post‐ICH and the proteins were transferred by electroblotting onto polyvinylidene fluoride membranes. The membranes were incubated with an anti‐CIRP goat polyclonal antibody (1:200, ab106230; Abcam), a rabbit polyclonal anti‐TLR4 antibody (1:500, ab13556; Abcam), and a mouse monoclonal anti‐NF‐κB p65 antibody (1:1,000) overnight at 4°C, respectively. Then, incubating membranes with HRP‐conjugated goat anti‐rabbit secondary antibodies (1:6,000, Zhongshan Golden Bridge Inc.) for 1.5 hr at room temperature. β‐actin was used as the control protein. By computer‐assisted image analyzing and densitometry scanning, the signals were quantified and the ratio of the value of detected CIRP protein band to the β‐actin band was the protein levels.

### 2.7 Fluoro Jade B (FJB) staining

Fluoro Jade B staining was measured as previously described methods (Yuan et al., 2019). Briefly, brain tissues were dehydrated in 15%–30% sucrose solutions and cut into 25 μm thick sections. Next, brain sections were immersed orderly in 1% sodium hydroxide in 80% alcohol for 10 min, 70% alcohol for 2 min, distilled water for 5 min, and 0.06% potassium permanganate for 10 min at room temperature and then washed with distilled water. Then, the sections were incubated in 0.01% FJB solution (Millipore) for 30 min. After washing with distilled water, the sections were dehydrated in gradient alcohol solutions and cleared in xylene, and finally, covered with coverslips in DPX (Sigma‐Aldrich). The brain tissues were examined by fluorescence microscopy (Olympus Microscope System BX51; Olympus). For quantification of positive FJB staining cells, three researchers calculated the number of positive cells in each randomized microscopic field independently from each mouse by using ImageJ (ver. 1.46J), and the results were the average values of numbers of each mouse.

### 2.8 Brain water content (BWC)

Brain water content was determined as previously described (Yuan et al., 2019). Briefly, mice at 1, 3, 5, and 7 days following ICH were deeply anesthetized, and the cerebral tissues were removed. Then, filter paper was used to blot the surface water on the cerebral tissues. The perihematomal brain tissues were immediately weighed to obtain the wet weight and then dried at 100°C for 24 hr to obtain the dry weight. BWC was calculated using the following formula: BWC (%) = (wet weight − dry weight)/wet weight × 100%.

### 2.9 Neurological deficient score (NDS)

The behavioral tests, including circling behavior, climbing, front limb symmetry, and body symmetry, were applied to characterize neurological deficits at different time points after ICH (Yuan et al., 2019). The scoring was completed by two trained investigators who were blinded to the group assignments of the mice.

### 2.10 Statistical analysis

All data are expressed as means ± *SD* or as a percentage, and analyses were performed with SPSS 13.0 software. A chi‐squared test was used to compare the differences in proportions between two groups. One‐way analysis of variance (ANOVA) test or Student's *t* test was used to compare two groups. *p*‐value < .05 was considered as significant differences.

## 3 RESULTS

### 3.1 Serum CIRP levels were increased in patients with ICH and in a positive correlation with neurologic deficits

**FIGURE 1:**
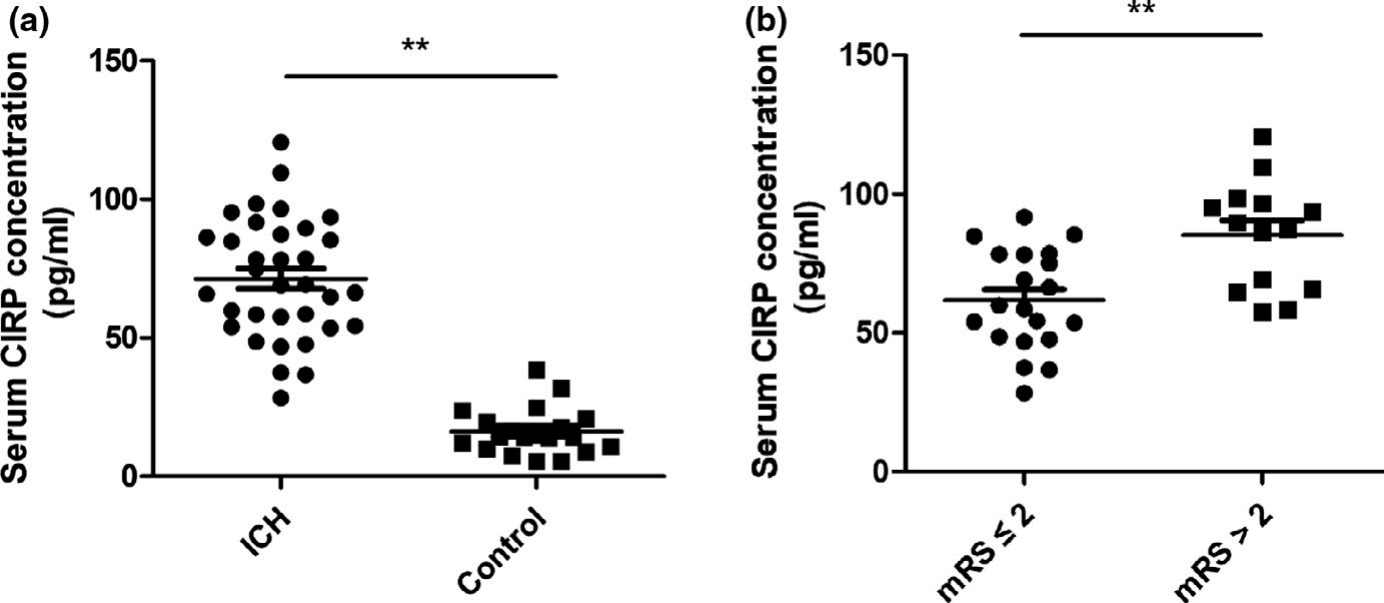
CIRP levels were increased in the blood of patients with ICH and positively correlated with neurologic deficits. (a) Serum CIRP levels in patients with ICH and control individuals were detected by ELISA kits. (b) The serum CIRP levels in patients with mRS > 2 were higher than that in mRS ≤ 2 at 3 months after ICH. **p < .01 versus the control individuals

The CIRP ELISA kits were used for the analysis of the expression of CIRP in blood among 34 ICH patients and 20 healthy controls (sex and age‐matched). Table 1 showed the comparison data of the baseline level of the two groups. When compared to the healthy controls, serum CIRP levels were significantly increased in the ICH patients (*p* < .01, Figure 1a). Furthermore, we found that among the ICH patients, the serum CIRP levels in mRS scores > 2 at 3 months after ICH were markedly higher than that in mRS scores ≤ 2 (*p* < .01, Figure 1b).

**TABLE 1:** General clinical features of ICH patients and healthy control group

	Control individuals (*n* = 20)	ICH patients (*n* = 34)	*p*‐value
Age, years	55.8 (8.43)	59.1 (9.18)	>.05
Male, %	55.0	52.9	>.05
Body temperature, °C	36.8 (0.62)	37.1 (0.58)	>.05
History of vascular risk factors, %			
Hypertension	80.0	85.3	>.05
Alcohol consumption	40.0	41.2	>.05
Smoking habit (current)	30.0	32.4	>.05
Diabetes	15.0	17.6	>.05
Laboratory parameters			
Leukocyte count, ×10^9^/L	7.08 (1.59)	7.41(1.68)	>.05
Serum glucose, mmol/L	6.21 (2.03)	6.34 (1.92)	>.05
Platelet count, ×10^9^/L	173 (41)	194 (54)	>.05
IL‐6, pg/ml	10.76 (4.5)	168.4 (57.3)	<.01
TNF‐a, pg/ml	31.5 (9.7)	115.2 (32.5)	<.01

Note

Values are presented as proportions or mean (*SEM*).

### 3.2 CIRP expression levels were markedly increased after ICH in mice

**FIGURE 2:**
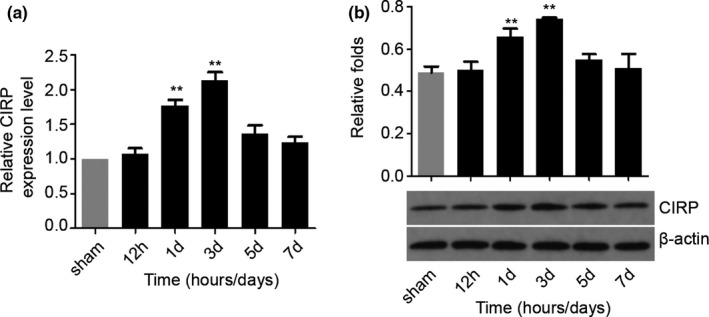
CIRP expression in the perihematomal brain tissues after ICH. (a) Real‐time PCR results showing *cirp* mRNA expression in the perihematomal brain tissues of mice at 12 hr to 7 days post‐ICH. (b) Representative bands and densitometric quantification of CIRP expression in the perihematomal brain tissues of mice at 12 hr to 7 days post‐ICH. *n* = 4 per group, **p < .01 versus the Sham groups

Next, we want to investigate the CIRP expression profiles in the perihematomal brain tissues after ICH by measuring CIRP protein and mRNA levels. First, we performed RT‐PCR to detect the changes in *Cirp* gene at different time points after ICH in the perihematomal brain tissues of mice. We noticed that compared with the sham groups, the *Cirp* mRNA expression increased significantly at 1 and 3 days after ICH (*p* < .01), while nonsignificantly increasing was found at 5 and 7 days post‐ICH (Figure 2a). To further testify the results of the RT‐PCR, Western blotting was adopted to detect the level of CIRP protein. We found that the CIRP protein level was increased markedly as well in the perihematomal brain tissues at the 1 and 3 days following ICH (*p* < .01, Figure 2b). These results indicate that CIRP may play critical roles in brain injury caused by ICH.

### 3.3 CIRP knockout alleviates inflammatory injury in perihematomal tissue after ICH

**FIGURE 3:**
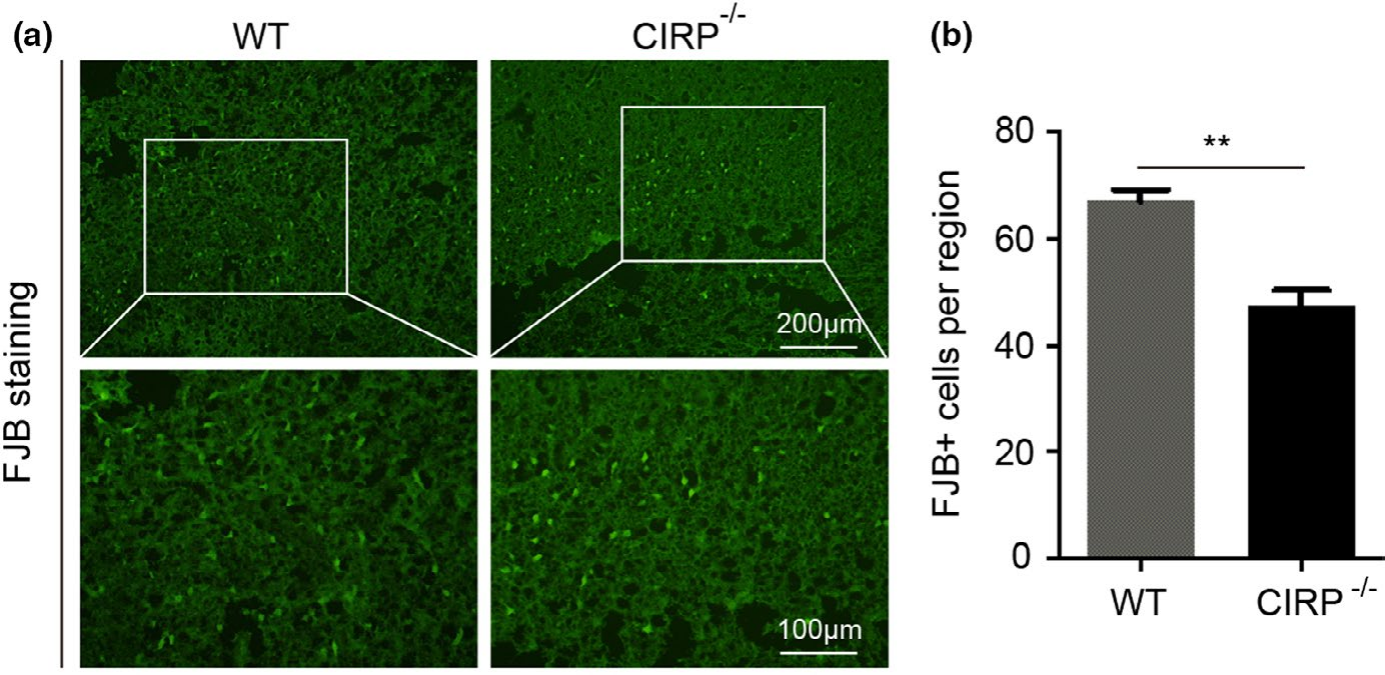
FJB staining of perihematomal brain tissues at 3 days post‐ICH. (a) Representative images of FJB staining between CIRP^−/−^ and WT mice after ICH. (b) Quantification of degenerated neurons by FJB staining in perihematomal brain tissues of CIRP^−/−^ and WT mice after ICH. *n* = 4 per group, **p < .01 versus the WT groups

**FIGURE 4:**
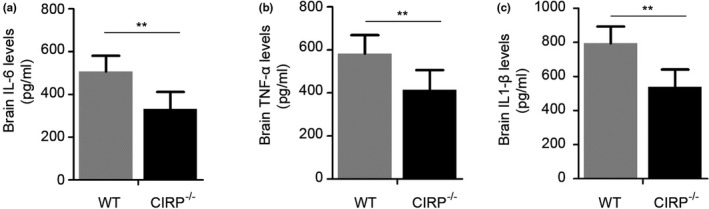
Levels of inflammatory factors in perihematomal brain tissues of mice. (a–c) The brain IL‐6 (a), TNF‐α (b), and IL‐1β (c) levels were detected by ELISA kits between CIRP^−/−^ and WT mice at 3 days post‐ICH. *n* = 4 per group, **p < .01

**FIGURE 5:**
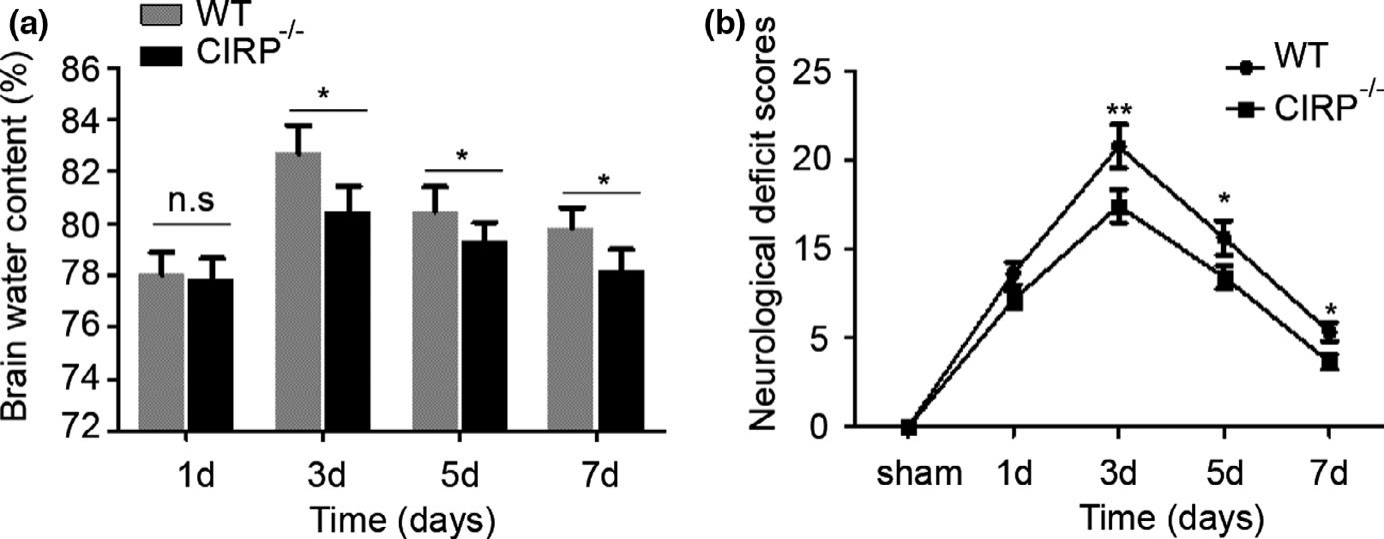
The brain water contents (BWC) and neurological deficit scores (NDS) were measured after ICH. (a) BWC in the WT and CIRP^−/−^ mice at 1, 3, 5, and 7 days after ICH (*n* = 4 per group, *p < .05) versus the CIRP^−/−^ group), n.s. indicates nonsignificance. (b) NDS in the WT and CIRP^−/−^ mice at 1, 3, 5, and 7 days after ICH (*n* = 9 per group, **p < .01 versus the CIRP^−/−^ group, *p < .05 versus the CIRP^−/−^ group)

Increased degenerated neurons, inflammatory factors, brain water contents (BWC), and worsen neurological deficit scores (NDS) of mice have also been found to involve in brain injury caused by ICH. In order to investigate the roles of CIRP in brain injury after ICH, we constructed the CIRP^−/−^ ICH mice. First, FJB staining was performed to examine changes in the numbers of degenerated neurons that existed in the perihematomal brain tissues at 3 days post‐ICH, and we found that the number of degenerated neurons in CIRP^−/−^ mice after ICH was less than that in WT mice (*p* < .01, Figure 3). Then, we used the ELISA kits to measure the inflammatory factors at 3 days after ICH and found that the levels of IL‐6, TNF‐α, and IL‐1β were markedly reduced in the perihematomal brain tissues of CIRP^−/−^ mice compared with the WT mice at 3 days after ICH (Figure 4). Given that BWC was one of important indices of brain injury caused by ICH, then we compared the BWC of perihematomal brain tissues at 1, 3, 5, and 7 days following ICH between CIRP deficiency mice and WT mice. We found that compared with the WT ICH group, the BWC around the perihematomal brain tissues was evidently reduced in CIRP^−/−^ mice on days 3, 5, and 7 (Figure 5a). In addition, NDS was used to study the biofunctional role of CIRP in ICH mice and the results showed that compared with the WT group, the NDS scores were remarkably decreased in CIRP^−/−^ group at 3, 5, and 7 days post‐ICH (Figure 5b). These data suggested that enhanced expression of CIRP aggravate the ICH‐induced brain injury.

### 3.4 CIRP increased inflammatory injury caused by ICH may via TLR4 signaling

**FIGURE 6:**
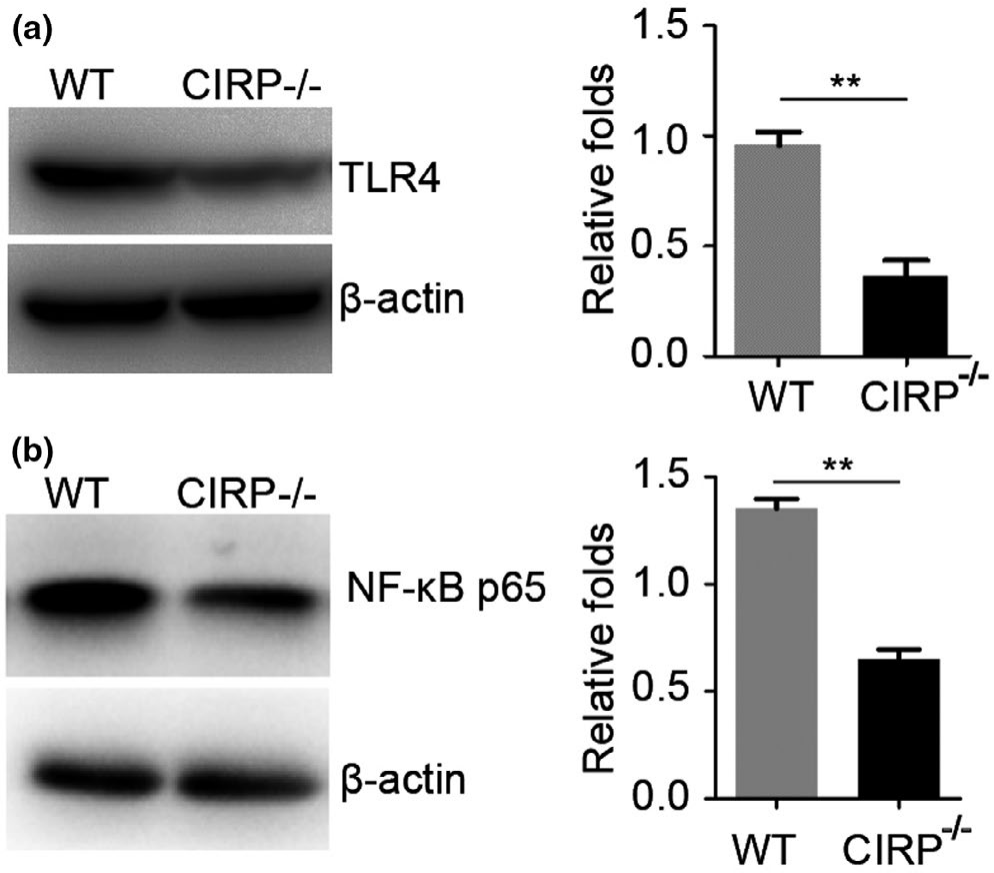
TLR4 and NF‐κB expression levels of perihematomal brain tissues at 3 days post‐ICH. (a, b) Representative bands and densitometric quantification of TLR4 (a) and NF‐κB p65 (b) expression in the perihematomal brain tissues at 3 days post‐ICH between WT and CIRP^−/−^ mice (*n* = 3 per group, **p < .01)

Then, we studied the potential mechanism of upregulated expression of CIRP induced brain injury after ICH. As CIRP was considered as a DAMP to involve in the activation of TLR4 to trigger and aggravate the inflammatory response (Aziz, Brenner, & Wang, 2019; Qiang et al., 2013). Therefore, we want to know whether the enhanced CIRP could influence the TLR4 signaling after ICH. Using the CIRP^−/−^ mice, we found that when compared with the WT mice, TLR4 expression was significantly decreased at 3 days following ICH (*p* < .01, Figure 6a). Additionally, we also detected the NF‐κB levels, which was a downstream signaling of TLR4, and found that the NF‐κB levels of perihematomal brain tissues at 3 days post‐ICH were also markedly decreased in CIRP knockout mice compared to WT mice (*p* < .01, Figure 6b). Therefore, these results suggest that increased CIRP after ICH in the perihematomal brain tissues could aggravate brain injury may via activating TLR4 signaling.

## 4 DISCUSSION

The upstream triggering mechanisms of inflammatory injury caused by intracerebral hemorrhage (ICH) remain to be elucidated. Given that TLR4 signaling has been shown as one of the important events to induce brain injury after ICH, and recently, CIRP was demonstrated as one of DAMPs. Therefore, by using the autologous ICH mice, we explored the role of CIRP in responding to a cerebral hemorrhagic insult. In this study, we first detected the upregulation of CIRP mRNA and protein expression in the perihematomal brain tissues of ICH mice. Then, we further validated the contribution of CIRP in ICH by evidencing that the FJB^+^ degenerated cells, brain water content (BWC), NDS, the proinflammatory cytokines expression in CIRP^−/−^ mouse brain are markedly reduced in comparison with that of wild type ICH models. Thus, elevation of CIRP may be a therapeutic target for the treatment of brain injury during ICH.

A growing body of evidence indicates that neuroinflammation is the major reason that contributes to the brain injury induced by ICH (Yuan et al., 2019; Zhang et al., 2017). TLRs, as the extracellular innate immune sensors, have been shown to be responsible for receiving the extracellular signaling and activating the cells to induce inflammatory cascades (Fang, Wang, Zhou, Wang, & Yang, 2013). Although previous studies have already identified some DAMPs, like heme, hemoglobin (Hb), and HMGB1, which could trigger the TLRs signaling after ICH, there may be also exist some other DAMPs after ICH (Zhou, Wang, et al., 2014). Interestingly, recent studies have found that CIRP could act as a DAMP to activate inflammatory cells in a TLR4‐dependent manner (Li et al., 2017; Morf et al., 2012). Considering that DAMPs were important factors to activate inflammatory responses after ICH, therefore, in this study we investigate whether the CIRP also has important roles in regulating inflammation after ICH. Our research data showed that increased elevation of CIRP in the perihematomal brain tissues, which was similar to the other results in response to hypothermia and cerebral ischemia (Liu, Zhang, Li, & Xue, 2010; Zhou, Yang, et al., 2014), exacerbate the brain injury following ICH, which was in consistent with the previous reports investigated in the ischemic stroke (Aziz et al., 2019).

Cold‐inducible RNA‐binding protein has been shown to be expressed in several tissue types, and with the highest expression level of CIRP mRNA in the testis (Xue et al., 1999; Zhou, Zheng, Yang, Zhang, & Chen, 2009). CIRP mRNA expression detected by northern blot analysis also showed that brain could express the CIRP, which did not differ markedly among the brain regions, such as cortex, hippocampus, and striatum (Liu et al., 2010; Xue et al., 1999). Additionally, in situ hybridization histochemistry method revealed that the expression of CIRP in neurons also existed in the hippocampus and the cerebral cortex of normal rat brain (Xue et al., 1999). In our study, we also detected the CIRP mRNA and proteins in brain tissues, and found that the CIRP expression was significantly increased after ICH. However, it is a pity that we did not investigate the cellular source of increased expression of CIRP after ICH in this study, as one study has shown that microglia‐derive BV2 cell lines were the major effect cells for CIRP function for neuroinflammation (Zhou, Yang, et al., 2014).

Additionally, using the clinical ICH patients' serum samples, we detected the CIRP levels and found that serum CIRP expression levels were significantly increased in ICH patients in comparison with the control individuals. Furthermore, we want to know whether the increased serum CIRP levels were also correlated with the prognosis of patients. Then, we divided these ICH patients into two groups according to their mRS at 3 months after ICH and found that the poorer outcome patients had higher serum CIRP levels compared to the good outcome group. Thus, like the previous study in the shock and sepsis showed (Morf et al., 2012), serum CIRP may also have the potential as an indicator for the prognosis after ICH. However, are still large works need to be done to demonstrate whether serum CIRP could act as a prognostic indicator.

In conclusion, we have demonstrated the expression and biological roles of CIRP in perihematomal brain tissue after ICH at the first time. The significant upregulation of CIRP expression in perihematomal brain tissues may be resulted in activation of TLR4 signaling to exacerbate ICH‐induced brain injury, which implying that CIRP may be a novel therapeutic target to reduce the disability caused by ICH.

## CONFLICT OF INTEREST

The authors have declared no conflict of interest.

## AUTHOR CONTRIBUTIONS

YZ conceived the idea, wrote the grant application, and secured the funding. YZ, KZ, and SC wrote/edited the manuscript. KZ, SC, WD, and JZ conducted the experiments. JH, LW, and ZG analyzed the data.

## Data Availability

The data that support the findings of this study are available from the corresponding author upon reasonable request.

## References

[brb31618-bib-0001] Aziz, M. , Brenner, M. , & Wang, P. (2019). Extracellular CIRP (eCIRP) and inflammation. Journal of Leukocyte Biology, 106(1), 133–146. 30645013 10.1002/JLB.3MIR1118-443R PMC6597266

[brb31618-bib-0002] Bolognese, A. C. , Sharma, A. , Yang, W. L. , Nicastro, J. , Coppa, G. F. , & Wang, P. (2018). Cold‐inducible RNA‐binding protein activates splenic T cells during sepsis in a TLR4‐dependent manner. Cellular & Molecular Immunology, 15(1), 38–47. 27569563 10.1038/cmi.2016.43 PMC5827170

[brb31618-bib-0003] Fang, H. , Wang, P. F. , Zhou, Y. , Wang, Y. C. , & Yang, Q. W. (2013). Toll‐like receptor 4 signaling in intracerebral hemorrhage‐induced inflammation and injury. Journal of Neuroinflammation, 10, 27. 23414417 10.1186/1742-2094-10-27 PMC3598479

[brb31618-bib-0004] Guide for the Care and Use of Laboratory Animals, 8th edition (2011). http://grants.nih.gov/grants/olaw/Guide‐for‐the‐care‐and‐use‐of‐laboratory‐animals.pdf

[brb31618-bib-0005] Li, Z. , Fan, E. K. , Liu, J. , Scott, M. J. , Li, Y. , Li, S. , … Fan, J. (2017). Cold‐inducible RNA‐binding protein through TLR4 signaling induces mitochondrial DNA fragmentation and regulates macrophage cell death after trauma. Cell Death & Disease, 8(5), e2775. 28492546 10.1038/cddis.2017.187 PMC5584526

[brb31618-bib-0006] Lin, S. , Yin, Q. , Zhong, Q. , Lv, F. L. , Zhou, Y. , Li, J. Q. , … Yang, Q. W. (2012). Heme activates TLR4‐mediated inflammatory injury via MyD88/TRIF signaling pathway in intracerebral hemorrhage. Journal of Neuroinflammation, 9, 46. 22394415 10.1186/1742-2094-9-46 PMC3344687

[brb31618-bib-0007] Liu, A. , Zhang, Z. , Li, A. , & Xue, J. (2010). Effects of hypothermia and cerebral ischemia on cold‐inducible RNA‐binding protein mRNA expression in rat brain. Brain Research, 1347, 104–110. 20546708 10.1016/j.brainres.2010.05.029

[brb31618-bib-0008] Morf, J. , Rey, G. , Schneider, K. , Stratmann, M. , Fujita, J. , Naef, F. , & Schibler, U. (2012). Cold‐inducible RNA‐binding protein modulates circadian gene expression posttranscriptionally. Science, 338(6105), 379–383. 22923437 10.1126/science.1217726

[brb31618-bib-0009] Ohnishi, M. , Katsuki, H. , Fukutomi, C. , Takahashi, M. , Motomura, M. , Fukunaga, M. , … Akaike, A. (2011). HMGB1 inhibitor glycyrrhizin attenuates intracerebral hemorrhage‐induced injury in rats. Neuropharmacology, 61(5–6), 975–980. 21752338 10.1016/j.neuropharm.2011.06.026

[brb31618-bib-0010] Qiang, X. , Yang, W. L. , Wu, R. , Zhou, M. , Jacob, A. , Dong, W. , … Wang, P. (2013). Cold‐inducible RNA‐binding protein (CIRP) triggers inflammatory responses in hemorrhagic shock and sepsis. Nature Medicine, 19(11), 1489–1495. 10.1038/nm.3368 PMC3826915 24097189

[brb31618-bib-0011] The 4th National Conference on Cerebrovascular Diseases (1996). Assessment of clinical nerve function defects in the patients with cerebral apoplexy. China Journal of Neurology, 29(6), 381.

[brb31618-bib-0012] Wang, Y. C. , Zhou, Y. , Fang, H. , Lin, S. , Wang, P. F. , Xiong, R. P. , … Yang, Q. W. (2014). Toll‐like receptor 2/4 heterodimer mediates inflammatory injury in intracerebral hemorrhage. Annals of Neurology, 75(6), 876–889. 24752976 10.1002/ana.24159

[brb31618-bib-0013] Xiong, X. Y. , Liu, L. , Wang, F. X. , Yang, Y. R. , Hao, J. W. , Wang, P. F. , … Yang, Q. W. (2016). Toll‐like receptor 4/MyD88‐mediated signaling of hepcidin expression causing brain iron accumulation, oxidative injury, and cognitive impairment after intracerebral hemorrhage. Circulation, 134(14), 1025–1038. 27576776 10.1161/CIRCULATIONAHA.116.021881

[brb31618-bib-0014] Xiong, X. Y. , Liu, L. , & Yang, Q. W. (2016). Functions and mechanisms of microglia/macrophages in neuroinflammation and neurogenesis after stroke. Progress in Neurobiology, 142, 23–44. 27166859 10.1016/j.pneurobio.2016.05.001

[brb31618-bib-0015] Xue, J. H. , Nonoguchi, K. , Fukumoto, M. , Sato, T. , Nishiyama, H. , Higashitsuji, H. , … Fujita, J. (1999). Effects of ischemia and H2O2 on the cold stress protein CIRP expression in rat neuronal cells. Free Radical Biology and Medicine, 27(11–12), 1238–1244. 10641716 10.1016/s0891-5849(99)00158-6

[brb31618-bib-0016] Yang, H. , Wang, H. , Shivalila, C. S. , Cheng, A. W. , Shi, L. , & Jaenisch, R. (2013). One‐step generation of mice carrying reporter and conditional alleles by CRISPR/Cas‐mediated genome engineering. Cell, 154(6), 1370–1379. 23992847 10.1016/j.cell.2013.08.022 PMC3961003

[brb31618-bib-0017] Yang, Y. R. , Xiong, X. Y. , Liu, J. , Wu, L. R. , Zhong, Q. , Zhou, K. , … Yang, Q. W. (2017). Mfsd2a (major facilitator superfamily domain containing 2a) attenuates intracerebral hemorrhage‐induced blood‐brain barrier disruption by inhibiting vesicular transcytosis. Journal of the American Heart Association, 6(7), e005811. 28724654 10.1161/JAHA.117.005811 PMC5586300

[brb31618-bib-0018] Yuan, J. J. , Zhang, Q. , Gong, C. X. , Wang, F. X. , Huang, J. C. , Yang, G. Q. , … Yang, Q. W. (2019). Young plasma ameliorates aging‐related acute brain injury after intracerebral hemorrhage. Bioscience Reports, 39(5), BSR20190537. 31040201 10.1042/BSR20190537 PMC6522807

[brb31618-bib-0019] Zhang, Z. , Zhang, Z. E. , Lu, H. , Yang, Q. , Wu, H. E. , & Wang, J. (2017). Microglial polarization and inflammatory mediators after intracerebral hemorrhage. Molecular Neurobiology, 54(3), 1874–1886. 26894396 10.1007/s12035-016-9785-6 PMC4991954

[brb31618-bib-0020] Zhou, K. W. , Zheng, X. M. , Yang, Z. W. , Zhang, L. , & Chen, H. D. (2009). Overexpression of CIRP may reduce testicular damage induced by cryptorchidism. Clinical & Investigative Medicine, 32(2), E103–E111. 19331798 10.25011/cim.v32i2.6027

[brb31618-bib-0021] Zhou, M. , Yang, W. L. , Ji, Y. , Qiang, X. , & Wang, P. (2014). Cold‐inducible RNA‐binding protein mediates neuroinflammation in cerebral ischemia. Biochimica et Biophysica Acta, 1840(7), 2253–2261. 24613680 10.1016/j.bbagen.2014.02.027 PMC4061249

[brb31618-bib-0022] Zhou, Y. , Wang, Y. , Wang, J. , Anne Stetler, R. , & Yang, Q. W. (2014). Inflammation in intracerebral hemorrhage: From mechanisms to clinical translation. Progress in Neurobiology, 115, 25–44. 24291544 10.1016/j.pneurobio.2013.11.003

[brb31618-bib-0023] Zhu, X. , Buhrer, C. , & Wellmann, S. (2016). Cold‐inducible proteins CIRP and RBM3, a unique couple with activities far beyond the cold. Cellular and Molecular Life Sciences, 73(20), 3839–3859. 27147467 10.1007/s00018-016-2253-7 PMC5021741

